# Linear Versus Curved Incision for Transcutaneous Bone Conduction Implants: Comparative Scar Assessment

**DOI:** 10.1055/a-2852-9524

**Published:** 2026-08-14

**Authors:** Leonardo Elías Ordóñez-Ordóñez, José Agustín Caraballo-Arias, Hernán Díaz-Badillo, Paola Andrea Medina-Bravo, Camilo Macias-Tolosa, Jorge Alexis Medina-Parra, Esther Sofía Angulo-Martínez, Estefany Catherine Hernández

**Affiliations:** 1 Department of Otolaryngology, Clínica Universitaria Colombia, Clínicas Colsanitas SA, Keralty, Colombia; 2 Department of Otolaryngology, Facultad de Medicina, Fundación Universitaria Sanitas (Unisanitas), Keralty, Colombia; 3 Hospital Militar Central, Universidad Militar Nueva Granada, Bogotá, Colombia; 4 Anaboleas Research Team, Endorsed by Clínica Universitaria Colombia and Fundación Universitaria Sanitas (Unisanitas). Recognized by Colciencias (2024) Ministry of Science, Technology and Innovation of Colombia, Colombia; 5 Department of Otolaryngology, Clínica Los Nogales, Bogotá, Colombia

**Keywords:** bone conduction implant, patient-reported outcomes, patient and observer scar assessment scale, conductive hearing loss, mixed hearing loss

## Abstract

**Introduction:**

In some patients with conductive or mixed hearing loss, or single-sided deafness, bone conduction implants (BCIs) offer an effective treatment; and scar appearance is a relevant factor in the overall outcome.

**Objective:**

To compare scar appearance and alopecia between linear and curved scalp incisions in patients undergoing transcutaneous BCIs surgery.

**Methods:**

An observational, cross-sectional study was conducted at a tertiary referral center in patients who underwent transcutaneous BCI. Postoperative follow-up period was between 6m to 3y. Scar evaluation was performed using the Patient and Observer Scar Assessment Scale (POSAS) and the Vancouver Scar Scale (VSS). Alopecia was defined as absence of hair follicles within 2 mm of the incision line. Statistical comparisons between linear and curved incisions groups were conducted, as well as multivariate analysis with logistic regression models.

**Results:**

Eighty-four cases were analyzed: 28 underwent linear incisions and 56 underwent curved incisions. Patients reported significantly better scar appearance with linear incisions in POSAS, *p* = 0.035. Alopecia occurred less frequently among patients who received the Bonebridge implant (OR = 0.113; 95% CI: 0.027–0.478; *p* = 0.003). No statistically significant differences were found in observer assessment of POSAS or VSS scores.

**Conclusion:**

Linear incisions may offer advantages in scar appearance with transcutaneous BCIs. Certain scar characteristics may also be influenced by the type of device and the surgical approach required for its placement, making it challenging to isolate this effect from that attributed solely to type of incision. These results highlight the need to consider both cosmetic factors and auditory outcomes in surgical planning.

## Introduction

Hearing impairment, regardless of etiology, significantly impacts quality of life by interfering with verbal communication, language development, education, and occupational performance. According to the World Health Organization, 1 over 1.5 billion people globally experience some degree of hearing loss during their lifetime, with approximately 430 million requiring specialized medical intervention.

While conventional acoustic hearing aids remain the primary method of auditory rehabilitation, certain patients—such as those with microtia or a canal wall-down mastoidectomy—do not benefit adequately from them or cannot use them at all. In such cases, bone conduction devices (BCDs) offer an effective alternative for individuals with conductive hearing loss (CHL), mixed hearing loss (MHL), or single-sided deafness (SSD). 2 3 4 5 6

Among BCDs, implantable devices (bone conduction implants, BCIs) generally offer superior auditory outcomes compared with non-surgical systems. BCIs are categorized by the type of vibration transmission (passive or active) and the interface with the sound processor (percutaneous or transcutaneous). 3 6 Passive BCIs rely on an external processor that converts sound into vibrations, which are transmitted to the cochlea via the skull (e.g., Sophono, Baha Connect, Ponto). In contrast, active BCIs use an internal transducer that directly generates mechanical vibrations in response to electrical input (e.g., Bonebridge, Osia). The connection may be percutaneous—via an abutment that penetrates the skin (e.g., Baha Connect, Ponto)—or transcutaneous via magnetic coupling through intact skin (e.g., Sophono, Bonebridge, Baha Attract, Osia). 3 5 7

Despite excellent auditory performance, percutaneous devices are associated with higher rates of skin complications such as inflammation, infection, and skin overgrowth. 2 3 Transcutaneous systems were developed to minimize these risks, 3 4 although they may cause discomfort due to magnetic pressure and raise cosmetic concerns, particularly regarding scarring and alopecia. Among transcutaneous systems, Sophono was the first to be developed, transmitting vibrations through intact skin, is placed by a semicircular retroauricular incision at a 45° angle, 55–60 mm from the external auditory canal. 3 Introduced in 2013, Baha Attract aimed to reduce complications associated with percutaneous systems. It employs a titanium osseointegrated implant with an internal magnet, inserted through a C-shaped incision located 15 mm from the magnet site and 50–70 mm from the auditory canal. 3 The Bonebridge system requires a semicircular retroauricular incision with at least a 5 mm margin to minimize extrusion risks. 8 9 The most recent FDA-approved system, Osia (2019), involves a semicircular retroauricular incision positioned 10–15 mm anterior or posterior to a surgical template, adjusted to auricular anatomy. 7

Regarding transcutaneous devices, linear incisions have recently been proposed as an alternative to traditional semicircular ('C-shaped') incisions, offering reduced soft tissue trauma, shorter surgical times, fewer postoperative complications, and improved cosmetic outcomes. 10 11 However, the choice of surgical incision depends on optimal surgical exposure, the patient's anatomy, and the manufacturer's recommendations for each device. 7 8 9 Studies in hair-bearing regions, such as reconstructive brow surgery, describe the use of 20-45 degree beveled incisions to preserve hair follicles and promote postoperative hair regrowth, even if hair shafts are transected. 12 Hair follicle preservation techniques are important in surgical procedures involving scalp areas for better postoperative hair regrowth.

Aesthetic considerations, particularly scar visibility and alopecia, are important to patient satisfaction and perceived surgical success, especially in procedures involving exposed or hair-bearing areas. Therefore, both functional and cosmetic results must be considered in the evaluation of bone conduction device implantation, given their combined effect on quality of life. 13 It is therefore important to understand which type of incision may offer better aesthetic outcomes in BCI surgery. To give greater clarity to this issue, the present study was performed to assess a comparative evaluation of linear and curved incisions regarding scar appearance, as assessed by both the surgeon and the patient, as well as their association with alopecia. The present study aimed to test whether linear incisions lead to less noticeable scarring.

## Methods

### Study Design

This was an observational study conducted in patients who received transcutaneous BCIs at a tertiary referral center in Bogota, Colombia.

The study complies to the ethical principles outlined in the Declaration of Helsinki, 14 as well as Colombian Resolution 8430 of 1993 for health research on human beings, 15 ensuring adherence to ethical standards and patient confidentiality. The study was approved by the Institutional Ethics Committee (CEIFUS 2255–21, act 043–21). The present study adheres to the STROBE (The Strengthening the Reporting of Observational Studies in Epidemiology) recommendations for reporting observational studies. 16

### Study Population and Inclusion/Exclusion Criteria

All participants in the study were patients who underwent transcutaneous BCI procedures as part of their treatment and were identified through audiological programming records of the study center. The search and registration of the information spanned from December 2022 until May 2024. Patients who underwent transcutaneous BCI placement between December 2019 and November 2023 (i.e., within 6 months to 3 years before the search period) were eligible for inclusion. Those who consented to participate and provided signed informed consent were enrolled. Exclusion criteria included refusal to participate, previous hearing implant surgery, incision in a hairless area (i.e., retroauricular area), prior procedures in the temporoparietal region, dermatological conditions affecting the healing process, and pre-existing baldness or seborrheic dermatitis. Demographic, clinical, and surgical data were obtained from the electronic medical records of the study center.

### Surgical Procedures

**Fig. 1:**
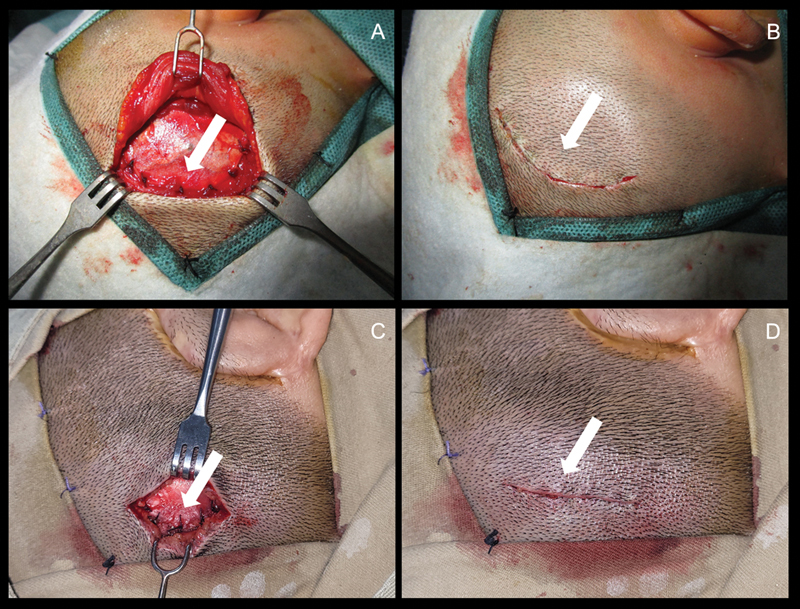
**Intraoperative images of patients with curved incision and linear incision.** The top panel ( **a, b** ) illustrates a case in which a curved incision was utilized for the placement of a Sophono implant. ( **A** ) depicts the closure of the musculoperiosteal plane using absorbable Vicryl 3–0 sutures, while ( **B** ) demonstrates the skin closure, performed with simple inverted Vicryl 3–0 stitches. Notably, the curved incision transects hair follicles at multiple points. The bottom panel (C, D) presents a case involving the placement of an Osia device using a linear incision. ( **C** ) shows the closure of the musculo-periosteal plane, and ( **D** ) depicts the skin closure, both completed with absorbable Vicryl 3–0 stitches. The orientation of the linear incision seeks to minimize disruption of hair follicles.

All surgeries were performed at the study center following standardized surgical techniques and manufacturer recommendations for each device. 3 7 8 17 Briefly, as a general concept applicable to all surgeries, under general anesthesia, with strict aseptic and antiseptic measures to minimize the risk of surgical site infections and delayed-onset biofilm-related infections, a curved or linear incision was made, followed by layered dissection to expose the skull at the selected site for device placement. Following this, the procedural steps for each device were executed, ensuring rigorous irrigation during bone drilling to minimize thermal trauma. The surgical wound was meticulously closed in two distinct layers: the musculoperiosteal layer utilizing simple sutures, followed by closure of the skin layer with simple subdermal sutures. Absorbable sutures (Vicryl 4–0 for pediatrics and 3–0 for adults), were employed for both layers. Following suturing, a compressive dressing was applied and maintained for 24 to 48 hours. The sound processor was fitted two to four weeks postoperatively. Intraoperative images for both curved and linear incisions are shown in Fig. 1 .

**Fig. 2:**
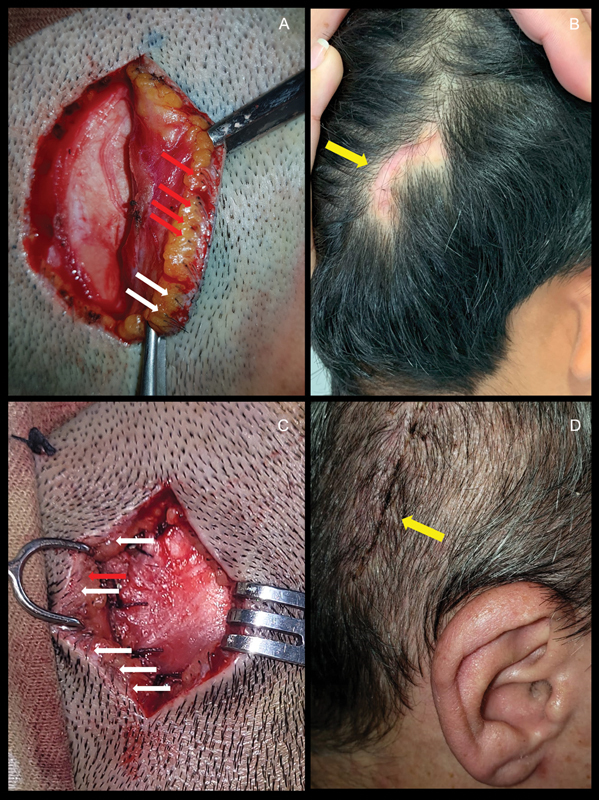
**Comparison between a curved incision and a linear incision on the scalp.** The top panel ( **A, B** ) illustrates a case in which a curved incision was utilized. ( **A** ) shows the intraoperative view, and ( **B** ) shows the scar state. The bottom panel ( **C, D** ) depicts a patient who underwent a linear incision. ( **C** ) shows the intraoperative view, and ( **D** ) the scar state. White arrows show preserved hair follicles, red arrows transected hair follicles and yellow arrows the final scar state.

In every procedure, efforts were made to minimize trauma to soft tissues performing only the necessary dissection. Additionally, the use of the bipolar/monopolar electrosurgical unit was reduced to the greatest extent possible. For linear incisions, careful orientation of the scalpel blade was employed to align it parallel to the hair follicles, thereby preserving hair bulb integrity and minimizing the risk of follicular transection. In contrast, curved incisions inherently pose a challenge due to their orientation, making follicular transection unavoidable in certain segments of the incision, Fig. 2 .

The choice of incision was determined by each surgeon on a case-by-case basis, aiming to provide adequate exposure for device placement while minimizing scarring. For Sophono and BAHA Attract, the incision was almost exclusively curved due to their design. 3 In Bonebridge cases, curved incisions were initially used, 8 9 but with time and growing experience, linear incisions became preferred. For Osia, being a relatively new device with limited early experience, surgeons generally followed the manufacturer's recommendation for curved incisions. 7

### Scar Assessments

The main outcome of the study was the evaluation of scars using the Patient and Observer Scar Assessment Scale (POSAS) and the Vancouver Scar Scale, along with an assessment of alopecia. These evaluations were performed on patients who were between six months and three years after their surgery, all measurements were made during a single session by a member of the research team (HDB, PAMB), under direct vision with good lighting conditions.

### Patient and Observer Scar Assessment Scale

POSAS is a tool used to assess scars from both the observer's (physician's) and the patient's perspective. It comprises two numerical scales: the Patient Scar Assessment Scale (P-SAS) and the Observer Scar Assessment Scale (O-SAS). The version used in this study was the one that had been cross-culturally adapted and validated for Latin American Spanish by Rodríguez et al. 18

The Observer Scale (O-SAS) comprises six characteristics of the scar, each one evaluated on an ordinal scale ranging from 1 (“similar to normal skin”) to 10 (“very different from normal skin”). The total score is obtained by summing all responses, yielding a final range of 6 to 60 points. Additionally, the scale includes a separate question assessing the overall opinion of the scar, which is not incorporated into the final score. The six characteristics evaluated were: vascularization, pigmentation, thickness, relief, pliability and surface area.

The Patient Scale (P-SAS) comprises seven questions, each rated on an ordinal scale from 1 to 10, with a total score ranging from 7 to 70 points. Two questions assess symptoms (pain and itching: 1 = No; 10 = Yes, very much), four evaluate specific parameters (color, stiffness, thickness, and surface area. 1 = No, like normal skin; 10 = Yes, very different), and one gauges the overall opinion of the scar (1 = similar to normal skin; 10 = very different from normal skin).

### Vancouver Scar Scale

The Vancouver Scar Scale (VSS) assesses four variables, each one rated on an ordinal scale: vascularity (0–3), height (0–3), pliability (0–5), and pigmentation (0–2). The total score, ranging from 0 to 13 points, is derived by summing the values assigned to each scar characteristic. 19 Originally developed for the assessment of burn scars, its application has expanded to include other types of scars, such as surgical scars. 20 This broader use makes it a valuable tool for comparing results across different studies.

### Assessment of Alopecia

Alopecia was defined as the absence of hair follicles within a 2-mm distance on either side of the incision scar. It was classified as a dichotomous variable (present/absent) and assessed through direct observation. In men and women with long hair, the hair was carefully parted to locate the scar, and for all the patients the evaluation was conducted under direct visualization with optimal lighting conditions, Fig. 2 .

### Statistical Analysis

A descriptive analysis was conducted using measures of central tendency and dispersion for quantitative variables (proportional and ordinals), while frequencies were reported for nominal variables. The Shapiro-Wilk test of normality was performed, and for normally distributed variables, parametric tests were used. Otherwise, nonparametric tests were applied under the assumption of ordinal variables. Stratified analyses were conducted to examine differences in demographic and clinical variables across groups categorized by incision type (curved versus linear) and age group (pediatric versus adult).

For the primary outcomes, the POSAS and VSS scales were analyzed as ordinal variables, with comparisons between incision groups (curved versus linear) performed using Wilcoxon rank-sum tests. The alopecia variable, a dichotomous variable, was compared across incision types using chi-square tests.

Multivariate modeling to adjust for potential confounders derived from stratified analyses were done using logistic regression models: ordinal logistic regression for ordinal outcomes (POSAS and VSS) and binary logistic regression for nominal outcomes (alopecia). Statistical significance was set at *p* < 0.05, and all statistical analyses were performed using SPSS version 26.0 (SPSS, Inc., Chicago, IL, USA).

## Results

**Fig. 3:**
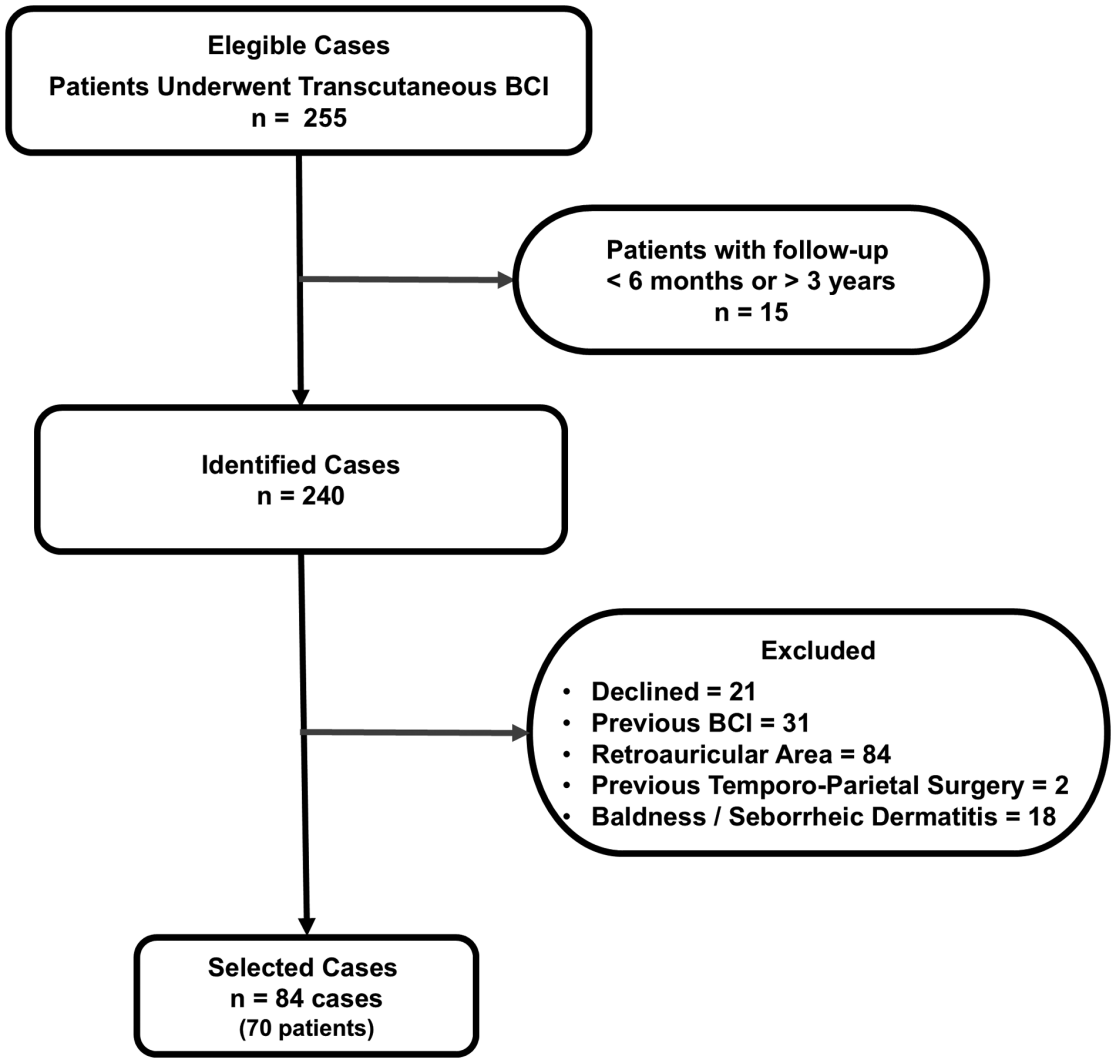
Flowchart of patient selection. BCI, bone conduction implant.

A total of 255 eligible cases were found for the study, of which 84 met the inclusion/exclusion criteria and were selected for the final analysis, Fig. 3 . The most common reason for exclusion was the incision being performed in a hairless area, specifically the retromastoid region ( *n* = 84), followed by cases involving patients with a previously implanted hearing device ( *n* = 31).

Demographics and clinical data of the patients in the study are presented in Table 1 . The mean age at the time of surgery was 26.02 ± 18.43 years for the total group, 8.58 ± 2.61 in pediatrics ( *n* = 25), and 35.71 ± 16.14 in the adult subgroup ( *n* = 45). 41 patients were male (58.6%), 14 patients underwent bilateral surgery (16.7%), and 52 cases (61.9%) underwent surgery in the right ear. The most common indication for surgery was CHL, observed in 65 patients (77.4%). Similarly, microtia accompanied by atresia or stenosis of the external auditory canal (EAC) was identified as the leading cause of hearing loss in 65 subjects also (77.4%). The most utilized devices were active transcutaneous implants, with OSIA being the most frequent in 50 cases (59.5%), followed by Bonebridge in 24(28.6%). Regarding the type of incision, curved incisions were performed in 56 cases (66.7%), while linear incisions were employed in 28 (33.3%), in a two-to-one ratio.

**Table 1:** Demographics and clinical data of population of the study

Variables	Value
Demographics*	
** Age (years), mean ± SD †**	26.02 ± 18.43
**Age group, mean ± SD**	
** Pediatrics (<14years), n** **=** **25**	8.58 ± 2.61
** Adults (≥14 years), n** **=** **45**	35.71 ± 16.14
**Sex, n(%)**	
** Male**	41(58.6%)
** Female**	29(41.4%)
**Bilateral surgery, n(%)**	
** Yes**	14(16.7%)
** No**	70(83.3%)
Clinical and surgical data**	
**Side of surgery, n(%)**	
** Right**	52(61.9%)
** Left**	32(38.1%)
**Indication, n(%)**	
** Conductive hearing loss**	65(77.4%)
** Mixed hearing loss**	15(17.9%)
** SSD**	4(4.8%)
**Etiology of hearing loss, n(%)**	
** Microtia with atresia/stenosis of EAC**	65(77.4%)
** Sequels of COM**	16(19%)
** Idiopathic sudden hearing loss**	3(3.6%)
**Type of Device, n(%)**	
** Osia**	50(59.5%)
** Bonebridge**	24(28.6%)
** BAHA Attract**	5(6%)
** Sophono**	5(6%)
**Type of incision, n(%)**	
** Linear**	28(33.3%)
** Curved**	56(66.7%)

SD, Standard deviation; SSD, Single sided deafness; EAC, External auditory canal; COM, Chronic otitis media; BAHA, Bone anchored hearing aid.

*Considering the number of subjects, n = 70.

**Considering the number of cases (ears), n = 84.

† Shapiro-Wilk test of normality, p < 0.001

Table 2 presents the results stratified by age group (pediatric versus adult) and incision type (linear versus curved) to assess potential baseline differences between the groups. Analysis of age groups and surgical indications revealed that cases of MHL and SSD occurred exclusively in adults. Regarding etiology, no cases of COM sequelae (0%) or idiopathic sudden hearing loss (0%) were observed in pediatric patients. Additionally, Bonebridge was predominantly implanted in adults (91.7%), while the OSIA was distributed similarly between the two age groups. Variations in BCI indications and hearing loss etiology account for the differences observed across age groups. The analysis of incision types revealed that curved incisions were more frequently performed in cases of CHL (75.4%), whereas linear incisions were predominant in cases of MHL at 66.7%. Furthermore, in Osia implantations, most incisions were curved (90%), whereas BoneBridge procedures primarily involved linear incisions (70.8%). A statistically significant difference also was found in follow-up duration between patients with linear incisions (17.47 ± 10.68 months) and those with curved incisions (12.49 ± 6.51 months), *p* = 0 *.010* .

**Table 2:** Stratified analysis between pediatrics vs adult and type of incision groups

	Age group			Type of Incision		
*Variables*	Pediatric (<14y)	Adult (≥14y)	*p* value	Linear	Curved	*p* value
Age group**, n(%)						
** ** Pediatric				7(22.6%)	24(77.4%)	*0.110* †
** ** Adult				21(39.6%)	32(60.4%)	
**Sex** * **, n(%)**						
** ** Male	19(36.5%)	33(63.5%)	*0.929* †	17(32.7%)	35(67.3%)	*0.874* †
** ** Female	12(37.5%)	20(62.5%)		11(34.4%)	21(65.6%)	
**Side of surgery** ** **, n(%)**						
** ** Right	23(44.2%)	29(55.8%)	*0.076* †	18(34.6%)	34(65.4%)	*0.751* †
** ** Left	8(25%)	24(75%)		10(31.3%)	22(68.8%)	
**Indication** ** **, n(%)**						
** ** Conductive hearing loss	31(47.7%)	34(52.3%)	***<0.001*** ††	16(24.6%)	49(75.4%)	***0.003*** ††
** ** Mixed hearing loss	0(0%)	15(100%)		10(66.7%)	5(33.3%)	
** ** SSD	0(0%)	4(100%)		2(50%)	2(50%)	
**Etiology of hearing loss** ** **, n(%)**						
** ** Microtia with atresia/stenosis of EAC	31(47.7%)	34(52.3%)	***<0.001*** ††	18(27.7%)	47(72.3%)	*0.092* ††
** ** Sequels of COM	0(0%)	16(100%)		9(56.3%)	7(43.8%)	
** ** Idiopathic sudden hearing loss	0(0%)	3(100%)		1(33.3%)	2(66.7%)	
Type of device**, n(%)						
** ** Osia	24(48%)	26(52%)	***0.002*** ††	5(10%)	45(90%)	***<0.001*** ††
** ** Bonebridge	2(8.3%)	22(91.7%)		17(70.8%)	7(29.2%)	
** ** BAHA Attract	2(40%)	3(60%)		1(20%)	4(80%)	
** ** Sophono	3(60%)	2(40%)		5(100%)	0(0%)	
**Follow-up (months), mean±SD**	12.93±8.22	14.86±8.51	*0.312* ‡	17.47±10.68	12.49±6.51	***0.010*** ‡

SD, Standard deviation; SSD, Single sided deafness; EAC, External auditory canal; COM, Chronic otitis media; BAHA, Bone anchored hearing aid.

* Considering the number of subjects, n= 70.

** Considering the number of cases (ears), n= 84.

† Chi-square test.

†† Fisher's exact test.

‡ Student's t-test.

The results of the bivariate analyses for the main outcomes are summarized in Table 3 , which compares the two incision types based on scar assessment results using the POSAS and VSS scales, as well as the presence of alopecia. Analysis of the P-SAS revealed that patients perceived the curved incision as more noticeable than the linear incision; *p* = 0 *.035* . Similarly, alopecia was more frequently associated with the curved incision (77.1%) compared with the linear incision (22.9%); *p* = 0.019. However, no significant differences were identified in the O-SAS evaluations, nor in the total score of the VSS or any of its measured variables; *p* *>* *0.05* .

**Table 3:** Comparative scar assessment between curved vs. linear incisions in transcutaneous bone conduction implants (n = 84)

Scar outcome	Type of incision		
	Curved (n = 56)	Linear (n = 28)	*p*
**POSAS—M (IQR)**			
** Observer (total value: 6-60)**	11(7)	9.5(5)	0.145 †
** Observer (overall assessment: 1-10)**	2(1)	2(2)	0.330 †
** Patient (total value: 7-70)**	15(16)	11(11)	** 0.035 †**
**Vancouver Scar Scale, M(IQR)**			
** Total value (0-13)**	3(4)	3(3)	0.298 †
** Vascularity (0-3)**	0(1)	0(2)	0.768 †
** Pigmentation (0-2)**	1(1)	1(2)	0.261 †
** Pliability (0-5)**	1(2)	2(1)	0.066 †
** Height (0-3)**	0(1)	0(1)	0.897 †
**Alopecia, n(%)**			
** Yes**	37(77.1%)	11(22.9%)	**0.019** ††
** No**	19(52.8%)	17(47.2%)	

POSAS, Patient and Observer Scar Assessment Scale; M, Median; IQR, interquartile range.

† Wilcoxon rank-sum test.

†† Chi-square test.

Table 4 presents correlation measures examining the potential impact of follow-up duration (time of scar assessment) on scar evaluation outcomes. An inverse relationship was observed between follow-up and the total score of the O-SAS (Spearman's rho = -0.242, *p* = 0 *.026* ), the P-SAS (Spearman's rho= -0.260, *p* = 0 *.017* ), and vascularity in the VSS (Spearman's rho= -0.354, *p* = 0 *.001* ).

**Table 4:** Correlations between follow-up and scar outcomes (n = 84)

Scar outcome	Follow-up (months)	
	Spearman's rho	*p*
**POSAS**		
** Observer (total value: 6-60)**	**-0.242**	**0.026**
** Observer (overall assessment: 1-10)**	-0.150	0.173
** Patient (total value: 7-70)**	**-0.260**	**0.017**
**Vancouver Scar Scale**		
** Total value (0-13)**	-0.123	0.267
** Vascularity (0-3)**	**-0.354**	**0.001**
** Pigmentation (0-2)**	-0.061	0.582
** Pliability (0-5)**	-0.038	0.733
** Height (0-3)**	-0.040	0.718

POSAS, Patient and Observer Scar Assessment Scale.

To account for potential confounding factors identified in the stratified results ( Table 2 ) and the significant correlations between follow-up duration and certain scar characteristics ( Table 4 ), we performed multivariate logistic regression analyses. These analyses assessed the impact of two key confounders—device type and follow-up time—on the primary association between incision type and scar evaluation (alopecia and P-SAS), for which statistically significant relationships were observed as shown in Table 3 . During assumption verification for the logistic regression models, categories with insufficient sample sizes were identified: Sophono ( *n* = 5) and BAHA Attract ( *n* = 5) within type of device variable. These categories were excluded, resulting in a final sample size of *n* = 74 for the logistic regression analyses. During evaluation of the correlation matrices to confirm the absence of multicollinearity among the three independent variables (type of incision, type of device, and follow-up time), all correlation coefficients were found to be below 0.7. Consequently, for each dependent variable, a model was constructed including these three independent variables. For the dependent variable alopecia, a dichotomous nominal variable, the model was developed using binary logistic regression. For the dependent variable P-SAS, an ordinal variable, the model was constructed using ordinal logistic regression. The results of these analyses are presented in Table 5 .

**Table 5:** Multivariate logistic regression analyses to compare scar assessment in transcutaneous bone conduction implants (n = 74)*

	Dependent variables					
	Alopecia † (0 = No, 1 = Yes)			P-SAS †† (total value: 7-70)		
Independent variables	B	p value	OR (CI 95%)	B	p value	OR (CI 95%)
**Type of incision** **1 = curved**	0.009	0.991	1.009 (0.228-4.457)	1.213	**0.037**	**3.364** **(1.079-10.488)**
**Type of device** **1 = Bonebridge**	-2.179	**0.003**	**0.113** **(0.027-0.478)**	-0.616	0.270	0.540 (0.181-1.614)
**Follow-up** **(months)**	0.015	0.733	1.015 (0.932-1.105)	-0.048	0.120	0.953 (0.896-1.013)

P-SAS, Patient Scar Assessment Scale; B, Regression Coefficient Beta; OR, Odds Ratio; 95% CI, 95% confidence interval.

*Sample size after removing levels with fewer than 15 cases of the variables included in the models.

† Binary logistic regression.

†† Ordinal logistic regression.

For alopecia, the model was statistically significant in the Omnibus test of model coefficients (Chi-square, *p* = 0.001), indicating that the independent variables significantly explained the outcome. The variance explained ranged from 19.9% (Cox and Snell's R ^2^ ) to 26.8% (Nagelkerke's R ^2^ ), with an overall classification accuracy of 74.3% (models are accepted when >50%). The Hosmer–Lemeshow goodness-of-fit test confirmed an adequate fit ( *p* = 0.381). The associations between alopecia and incision type ( *p* = 0.991) and follow-up time ( *p* = 0.733) were no longer significant; however, a strong association with device type persisted ( *p* = 0.003). Use of the Bonebridge device showed a strong inverse relationship with alopecia, with patients being 7.7 times less likely (inverse of OR = 1/0.013) to be classified as having alopecia compared with those using the Osia device (OR = 0.113, 95% CI: 0.027–0.478; *p* = 0.003).

The P-SAS model was also statistically significant (likelihood ratio test, *p* = 0.049). The goodness-of-fit test (Pearson) indicated an adequate fit ( *p* = 0.352), and the model explained 10% of the variance (Nagelkerke's pseudo-R ^2^ ). A curved incision was associated with a 3.3-fold higher likelihood of a negative P-SAS perception compared with a linear incision (OR = 3.364, 95% CI: 1.079–10.488; *p* = 0.037), whereas the effects of type of device and follow-up time were not significant ( *p* = 0.270 and *p* = 0.120, respectively).

Regarding postoperative complications, minor adverse events were observed in 3 of the 84 cases (3.6%). One patient developed a hematoma requiring needle drainage in the outpatient setting, which resolved without further intervention. Another patient experienced a surgical site infection, presenting with edema, erythema, and wound pain five days postoperatively. No fluid collection was detected, and empiric antibiotic therapy with dicloxacillin (500 mg orally every 6 hours for seven days) led to symptom resolution. A third patient reported pain and erythema in the antenna region after 12 months of device use, attributed to excessive magnet strength. Management involved discontinuation of the sound processor for seven days and subsequent reduction of magnet strength, after which the patient resumed device use without complications or recurrence. No major complications, such as device extrusion, hardware failure, or the need for revision surgery; were observed during the follow-up period.

## Discussion

BCIs are effective devices in auditory rehabilitation and have been shown to significantly improve quality of life for patients with CHL, MHL, and SSD. 3 4 21 22 Over time, the design of these implants and the possible surgical techniques have evolved, aiming to minimize skin-related complications and improve soft tissue healing. One of these surgical issues has been the development of less invasive surgical approaches, including changes in the shape and orientation of scalp incisions. 10 11 Early experiences with linear incisions in patients receiving percutaneous BCI, such as the single vertical 23 or horizontal incision, 24 suggested the possibility of achieving comparable device performance with improved cosmetic results and lower complication rates.

The present study is strengthened by its substantial sample size, comprising 70 patients and 84 ears. Additionally, the use of validated scar assessment tools, such as POSAS and VSS, enhances the reliability of our findings. 18 20 The most relevant findings of our study were that linear incisions were associated with better patient-perceived scar outcomes (P-SAS) compared with curved incisions, and that a lower incidence of alopecia was observed among patients who received the Bonebridge implant ( Tables 3 and 5 ). Although the initial bivariate analysis ( Table 3 ) indicated that a linear incision was associated with a lower frequency of alopecia, the multivariate analysis using a binary logistic regression model ( Table 5 ) revealed that this lower frequency was linked to the Bonebridge implant, which was itself strongly associated with the use of a linear incision. Therefore, given that incision choice was closely tied to device type (linear incision with Bonebridge and curved incision with Osia), it is not easy to disentangle the positive or negative effects of these two variables on scar assessment.

Scalp alopecia is a notable postoperative concern for patients undergoing BCI implantation, especially in visible areas such as the temporoparietal scalp. This condition is directly related to the disruption or destruction of hair follicles during surgery and can be exacerbated by factors such as ischemia or excessive traction on scalp flaps. 12 25 When linear incisions are aligned and parallel to the natural orientation of hair follicles, this allows better preservation of these structures and may thus reduce the risk of permanent alopecia. These findings may be attributed in part to the preservation of hair follicles during surgery, as the orientation and type of scalp incision has been shown to directly impact follicular damage and subsequent alopecia. 12 25 To explain the effect of type of device on the presence of alopecia, this may relate to the design profile and placement of the implants. The Bonebridge device produces minimal protrusion on the skull surface, 8 9 whereas the Osia implant creates greater protrusion. 7 This prominence can exert tension along a perpendicular axis of the scar, resulting in scar widening and more evident alopecia.

Although some studies have supported the use of superiorly curved incisions, particularly for wide surgical exposure while minimizing cosmetic impact, 26 our results suggest that linear incisions, when carefully planned for hair follicular unit preservation, may offer superior scar outcomes in terms of patient-perceived quality.

Achieving better scar appearance can have meaningful effects on patient quality of life. 27 In BCI users, the postoperative scar remains a visible reminder of the procedure, and its aesthetic outcome may influence the patient's self-image, confidence, and overall acceptance of the device. Although most patients prioritize hearing improvement, the cosmetic impact of the surgery cannot be ignored. 10 28 Although specific studies on BCI are lacking, these findings suggest that minimizing scar visibility and alopecia through careful incision planning and selection of the device can improve patient satisfaction and quality of life.

Another relevant consideration is compatibility with device-specific surgical guidelines. While some devices, such as passive transcutaneous systems (e.g., Sophono, Baha Attract), recommend curved incisions, others, like Bonebridge allow or encourage linear incisions depending on anatomy and implant position. 11 29 Therefore, surgical planning must remain individualized, balancing optimal device performance with soft tissue preservation and aesthetic results. These findings may inform future design refinements of transcutaneous BCDs, favoring designs that minimize skull surface protrusion, as well as guide recommendations for related surgical techniques. An example of how clinical observations during device use have driven modifications in surgical technique can be seen in the evolution of the approach for BAHA Connect. Initially, placement was performed using a dermatome technique, later replaced by a linear incision with tissue reduction, then a linear incision without tissue reduction, and ultimately the punch technique (MIPS for the alternative brand Ponto). Each step progressively reduced complications related to skin integrity, infections, and extrusions; issues that were significant with percutaneous devices. 2 Transcutaneous BCIs may be undergoing a similar evolution in surgical techniques, particularly regarding alopecia and scar outcomes, where experience with linear incisions and device selection could drive changes in practice. For example, in our group, nearly all Osia procedures are now performed using a linear incision, based on the outcomes and experience we have observed.

Another relevant finding was the inverse correlation between follow-up duration and scar assessment scores, including vascularity, as perceived by both patients and clinicians (POSAS and VSS; Table 4) . This supports the notion that both objective and subjective scar quality improves with time, a finding consistent with prior scar maturation studies. Therefore, it is necessary to emphasize the need for long-term follow-up in scar assessment, as scar characteristics tend to evolve over time, and variability in follow-up durations may account for inconsistent findings across the literature. 30 This highlights the importance of long-term outcome assessments and may explain variability in existing literature based on follow-up duration. However, despite this natural progression, the initial appearance of the scar, particularly during the first year, remains clinically important for patient reassurance and satisfaction. 27 28

Our study presents several strengths, including a relatively large sample size and the use of culturally adapted, validated scar assessment tools. However, certain limitations must be acknowledged. The observational, cross-sectional design and non-randomized data may limit conclusions. In addition, scar evaluations were performed at varying postoperative time points rather than at a standardized follow-up interval. Another limitation was the close coupling of the most used devices with a particular incision, Bonebridge with linear incision and Osia with curved incision, which makes it difficult to separate the effects of the incision itself on the scar assessment from those that result from the device used. Additionally, the findings also reflect the experience of a single center with its corresponding surgical guidelines. Nevertheless, future multicenter longitudinal studies with larger sample sizes, seeking to control the effect of the type of device on the scar and extended follow-up periods (>5 years) are necessary to determine whether there are cosmetic and scar appearance benefits of a particular type of incision that persist over time.

## Conclusions

Our findings suggest that linear scalp incisions may offer superior scar outcomes in bone conduction implant surgery, particularly by improving scar characteristics from patient́s perceptions. A significant effect of the type of device on the presence of alopecia was also found, which could be partly explained by the protrusion of the device onto the surface of the skull. These benefits highlight the importance of incorporating aesthetic considerations into bone conduction hearing rehabilitation surgical planning. While this study provides valuable evidence, further prospective, multicenter research with long-term follow-up is needed to validate these findings and support future implant and technique development.
